# Trends in contraceptive method mix among adolescents and youth aged 15–24 in low- and middle-income countries

**DOI:** 10.3389/fgwh.2022.1061648

**Published:** 2023-01-11

**Authors:** Jane T. Bertrand, John A. Ross, Sydney R. Sauter

**Affiliations:** ^1^ Tulane University School of Public Health and Tropical Medicine, Department of International Health and Sustainable Development, New Orleans, LA, United States; ^2^ Independent Demographic Consultant, New Paltz, New York, United States

**Keywords:** family planning, method mix, method choice, youth, adolescents, method skew

## Abstract

**Background:**

Method mix – the percent distribution of contraceptive use by method among contraceptive users – reflects both client choice of method and method availability. In a country where clients have access to a wide range of methods at an affordable price, method mix is a strong proxy for method choice. In contrast, where access is limited by numerous factors – method availability, cost, or provider attitudes – method mix may not capture method choice well. Given that method mix can be measured reliably from population-based surveys, it is useful in exploring method choice. While the method mix for all women of reproductive age (15–49 years) has been described previously, the method mix for adolescents and young women aged 15–24 remains unexplored despite this population's high risk for unintended pregnancy.

**Objectives:**

This study investigates the contraceptive method mix for women aged 15–24 in low- and middle-income countries (LMICs) with national survey data and describes how the method mix differs by age group, geographic region, and marital status for women.

**Methods:**

Using data from the Demographic and Health (DHS) Surveys, the contraceptive method mix among women aged 15–24 across 64 LMICs is analyzed by age, marital status, and region, with measures of skew and average deviation. Three case studies are presented in which the trend over time in the method mix is examined.

**Results:**

There are large variations in method mixes across regions, which reflect their differences in various supply and demand constraints. However, there is consistently high usage of short-acting methods among both age groups, 15–19 and 20–24, compared to the full population of all women of reproductive age. Male condoms overwhelmingly predominate as the method used by women 15–24 in all regions.

**Conclusion:**

The marked differences found by marital status, region, and age show the need for programs to be tailored to local circumstances. Additionally, the large unmet need for contraception signals the ongoing urgency for strengthened programmatic efforts, and for a wider offering of methods to enlarge the choices available to young women. Unmarried women in particular deserve attention, as well as young married women who wish to postpone a pregnancy.

## Introduction

Method choice is a key component of effective international family planning programming that stresses client preferences and the ability of women to exercise free and informed choice when selecting a contraceptive method. According to the United States Agency for International Development, method choice exists when:

“client-centered information, counseling, and services enable women, youth, men, and couples to decide and freely choose a contraceptive method that best meets their reproductive desires and lifestyle, while balancing other considerations important to method adoption, use, and change” (1).

Method choice serves as an important indicator of the quality of care within family planning programs, as it reflects the ability of the program to meet client needs over time and across changing circumstances. By providing a full range of methods and comprehensive information, clients can identify a method that is available, acceptable, and suitable for their fertility needs (2). When method choice is limited, contraceptive use is impeded; when method choice is optimized, however, overall contraceptive adoption and continuation are increased among women with an unmet need for family planning (3). Thus, expanding contraceptive method choice is a key tool in improving family planning outcomes and ensuring the reproductive rights of women.

Method mix, also known as “method share,” is an indicator that reflects the distribution of contraceptive use among all contraceptive users. Method mix can be measured among all women of reproductive age currently using contraception or further analyzed by sub-groups found in population-based surveys. These sub-population analyses consider the various factors that may reflect changing life circumstances, including marriage, parity, and age. Trends over time in method mix reflect changing aspects of supply and demand.

While method choice pertains to the preferences of women in selecting a contraceptive method, method mix reflects the actual use of methods. On the supply side, method mix is influenced by several factors, including the availability of methods, stock-outs, cost barriers, geographic proximity, provider bias, management of side effects, and options for switching methods (4). In rural areas, for example, availability and usage of long-term methods such as the intrauterine device (IUD) may be curtailed due to the lack of provider knowledge and training (5–7). Moreover, the governments of some countries may not enable access to a full range of modern methods, and market trends can influence cost in the private sector. Costs can decline when donor agencies or national programs subsidize a method. Finally, the supply picture can change with the introduction of a new, low-cost method, as was the case with the injectable in the eastern and southern parts of sub-Saharan Africa (10, 11).

Factors affecting demand include those related to individual and societal attitudes regarding fertility and desired family size. The methods themselves have characteristics that can limit demand (e.g., a requirement for a vaginal procedure). At the individual level, concerns regarding health and known side effects are common reasons for non-use and discontinuation, as well as misconceptions surrounding contraception (9). Many women also cite fear of partner opposition as a major reason for non-use (10). Women are influenced by several factors at the societal level, including cultural beliefs, prevailing social norms, and stigma. Female sterilization, for example, is not widely used in many Middle Eastern countries, as the practice is considered in opposition to the faith teachings of Islam that equate the practice with the prohibited mutilation of the body (11). In contrast, female sterilization remains the most common method of contraception in Latin America, where the method has been widely accepted across the region (12). Other methods (such as male sterilization) remain unpopular across all regions and thus have little demand.

In a country with limited supply constraints, method mix is a strong indicator for method choice, as barriers to access are minimized or eliminated (6, 7). In contrast, where access is limited, method mix may not accurately capture method choice. Given that method mix can be measured reliably from population-based surveys, it is a useful tool in exploring method choice in the absence of a standard measure for this concept.

Method mix has been measured in two main ways. Bertrand et al. (13) first used the concept of method skew to define countries in which over 50% of total contraceptive use corresponded to a single method. Following this, follow-up studies across regions were conducted to analyze the long-term trends in method skew and potential correlations with various socioeconomic indicators and family planning interventions (4, 13, 14). Later, Ross et al. (16) proposed an alternative measure of “average deviation” (AD), in which the overall balance in the method mix is examined by the variation of method shares around their average.

Haakenstad et al. (17) in a 2019 analysis of method mix across 204 countries found that 28 countries demonstrated method skew among all women of reproductive age (15–49). However, previous research had estimated method skewness to be present in 34 countries during the same period (15). The reasons for the difference are unclear; the 2019 study uses technical methods that smooth extreme values, which may tend to soften disparities and bring some values below the 50% cutoff. In studies of skew among married women of reproductive age, the leading contraceptive method varies greatly across regions. The injectable, a short-term method, predominates in the method mix in sub-Saharan Africa, whereas long-term methods such as the intrauterine device (IUD) account for a great deal of method mix in the Asian region (15). These findings demonstrate the important influence of supply and demand across contexts (14, 16).

While the existing body of method mix research focuses on all women of reproductive age (15–49) in low-and middle-income countries (LMICs), research pertaining to method mix among youth and adolescents (aged 15–24) in LMIC s remains scarce. Youth and adolescents represent approximately 25% of the global population, and nearly 90% of these individuals reside in LMICs (18). While youth residing in LMICs constitute a large share of the global population, they also remain vulnerable to many poor sexual and reproductive health outcomes, including unintended pregnancy, unsafe abortion, and sexually transmitted infections (19). Roughly 10 million unintended adolescent pregnancies occur annually, and the estimated unmet need for contraception among adolescent girls in LMICs is much higher than that of all women in LMICs– 43% compared to 24%, respectively (20). Further, de Vargas Nunes Coll et al. (21) explored contraceptive prevalence and demand satisfied among adolescents aged 15–19 across marital status, parity, and region, finding that married adolescents with no children have the lowest contraceptive prevalence, and that the lowest demand for family planning satisfied by modern contraception among this group is in West and Central Africa. They noted the importance of these factors in assessing the needs of youth and adolescents; however, few studies exist to quantify the method choice of these individuals, and no study details the method mix of youth and adolescent women across both marital status and region.

Haakenstad et al. (17) have enlarged the evidence base concerning the persistently high unmet need among youth and adolescents globally. They have documented the method mix among women aged 15–24, noting the popularity of short-term methods including pills and condoms. However, their analysis did not account for the variations in method mix among youth and adolescents across countries or regions and did not explore the potential contextual factors that affect both method mix and method choice for these populations. In another report, Kalamar et al. (22) documented the persistently high non-use of contraception among youth and adolescents, though the type of method was not distinguished across regions or marital status. To assess the family planning environment for youth and adolescents across LMICs, a further investigation of the current method mix for this population is warranted. This analysis will inform efforts to expand access to a variety of contraceptive methods by youth and adolescents.

Thus, the objectives of the current study are to:

1. Present the current method mix for women aged 15–24 in LMICs with available survey data.
2. Determine how method mix differs by age group (aged 15–24 vs. 15–49, and aged 15–19 vs. 20–24).
3. Determine how method mix differs by geographic region and marital status for women aged 15–24.
4. Calculate the total number and percent of countries in which method skew is present among women aged 20–24.
5. Identify the predominant and runner-up methods among women aged 20–24.
6. Examine three select countries as case studies for the change over time in method mix among women aged 20–24.

## Materials and methods

### Data sources

The data employed here come from the Demographic and Health Surveys (DHS) conducted from 2000 onward in 64 LMICs, covering all married women aged 15–49, as well as unmarried sexually active women aged 15–24. These surveys are listed by region and year in Table 1. Other nationally representative surveys were not included in the analysis due to a lack of information on key variables. Regional averages use unweighted data, giving each country equal weight. For the results presented here that is preferable, since the use of population weights would generate results dominated by a small number of the largest countries. Thus, SSA is disproportionately represented in the global patterns of method mix reported in this article since most of the surveys are from this region and the analyses are unweighted. This is especially true for the unmarried sexually active women, as 70% of the countries with data on the group are in that region (Table 1). Detailed breakdowns by region for the group are not feasible due to the small number of countries with information, especially for the unmarried sexually active group.

**Table 1:** 64 DHS surveys by region.

ASIA		SUB-SAHARAN AFRICA, EAST AND SOUTH	
Afghanistan	2015	Angola	2015-16
Armenia	2015–16	Burundi	2016-17
Azerbaijan	2006	Comoros	2012
Bangladesh	2017–18	Eritrea	2002
Cambodia	2014	Eswatini	2006-07
India	2019–21	Ethiopia	2019
Indonesia	2017	Lesotho	2014
Maldives	2016–17	Malawi	2015-16
Myanmar	2015–16	Namibia	2013
Nepal	2016	Rwanda	2019-20
Pakistan	2017–18	South Africa	2016
Philippines	2017	Tanzania	2015-16
Timor-Leste	2016	Uganda	2016
Vietnam	2002	Zambia	2018
		Zimbabwe	2015
**CENTRAL ASIA REPUBLICS**			
Kyrgyz Republic	2012	**SUB-SAHARAN AFRICA, WEST AND CENTRAL**
Tajikistan	2017	Benin	2017–18
Turkmenistan	2000	Cameroon	2018
		Chad	2014–15
**LATIN AMERICA**		Congo	2011–12
Bolivia	2008	Congo Democratic Republic	2013–14
Colombia	2015	Cote d'Ivoire	2011–12
Dominican Republic	2013	Gabon	2012
Guatemala	2014–15	Gambia	2019–20
Guyana	2009	Guinea	2018
Haiti	2016–17	Liberia	2019–20
Honduras	2011–12	Mali	2018
Nicaragua	2001	Mauritania	2019–21
Peru	2012	Niger	2012
		Nigeria	2018
**MIDDLE EAST/NORTH AFRICA**		Sao Tome and Principe	2008–09
Egypt	2014	Senegal	2019
Jordan	2017–18	Sierra Leone	2019
Morocco	2003–04	Togo	2013–14
Turkey	2013		
Yemen	2013		

For brevity, regional acronyms are used: LA for Latin America, SSA-E for the East/South in sub-Saharan Africa, and SSA-W for the West/Central. Surveys in two regions – the Central Asia Republics (CAR) and the Middle East/North Africa (MENA), as well as in some additional countries – did not include data on unmarried sexually active women. For this population, the analysis is limited 38 countries with data for ages 15–19, and 41 countries (many overlapping) with data for ages 20–24.

### Data analysis

DHS survey data was analyzed using STATcomplier software (23). We examined the method mix for the eight methods of contraception in the DHS surveys that account for nearly all use: male and female sterilization, IUD, implant, pill, injectable, condom, and the traditional methods (rhythm and withdrawal). Emergency contraception was excluded from the analysis, given that most DHS surveys either omit it completely or report trivial usage.

Method skew was assessed in two ways: by identifying countries where one method accounts for 50% or more of the mix, and by calculating AD (average deviation) values for method balance. Use of the 8 methods adds to 100%, and the AD measure accounts for variations around the average of 12.5%. In a completely balanced mix, each method would have an equal share, at 12.5% each. The AD is calculated using the sum of the 8 absolute differences around the 12.5% average. Thus, the AD measure captures disparities in the mix, ranging from the lowest possible value of 0 (a perfectly balanced mix, never observed in practice) to higher values in which the mix is dominated by 1 or 2 major methods. The highest possible value is 22, in which a single method accounts for all use. Within the total range of 0 to 22, the higher the value the greater the disparity. There is no ideal value or mix pattern, but the AD affords a basis for comparing the imbalance of mixes across countries or over time.

Most results are displayed by region; however, for the unmarried sexually active group, Asia contains only two countries with the age breakdowns needed (India and the Philippines). Therefore, we have omitted Asia as a separate region in the figures but included those two countries in the results for “All Regions.” Given India's importance it is shown separately in Figures 2A,B.

Results are shown separately for the two age groups of 15–19 and 20–24. This separation is useful since the two groups represent women at distinctly different points in personal development. Where the patterns are similar between age groups, they are displayed only for the 20–24 age group, for simplicity. This is also the case in the text discussion of regional patterns for method skew and time trends.

## Results

**Figure 1:**
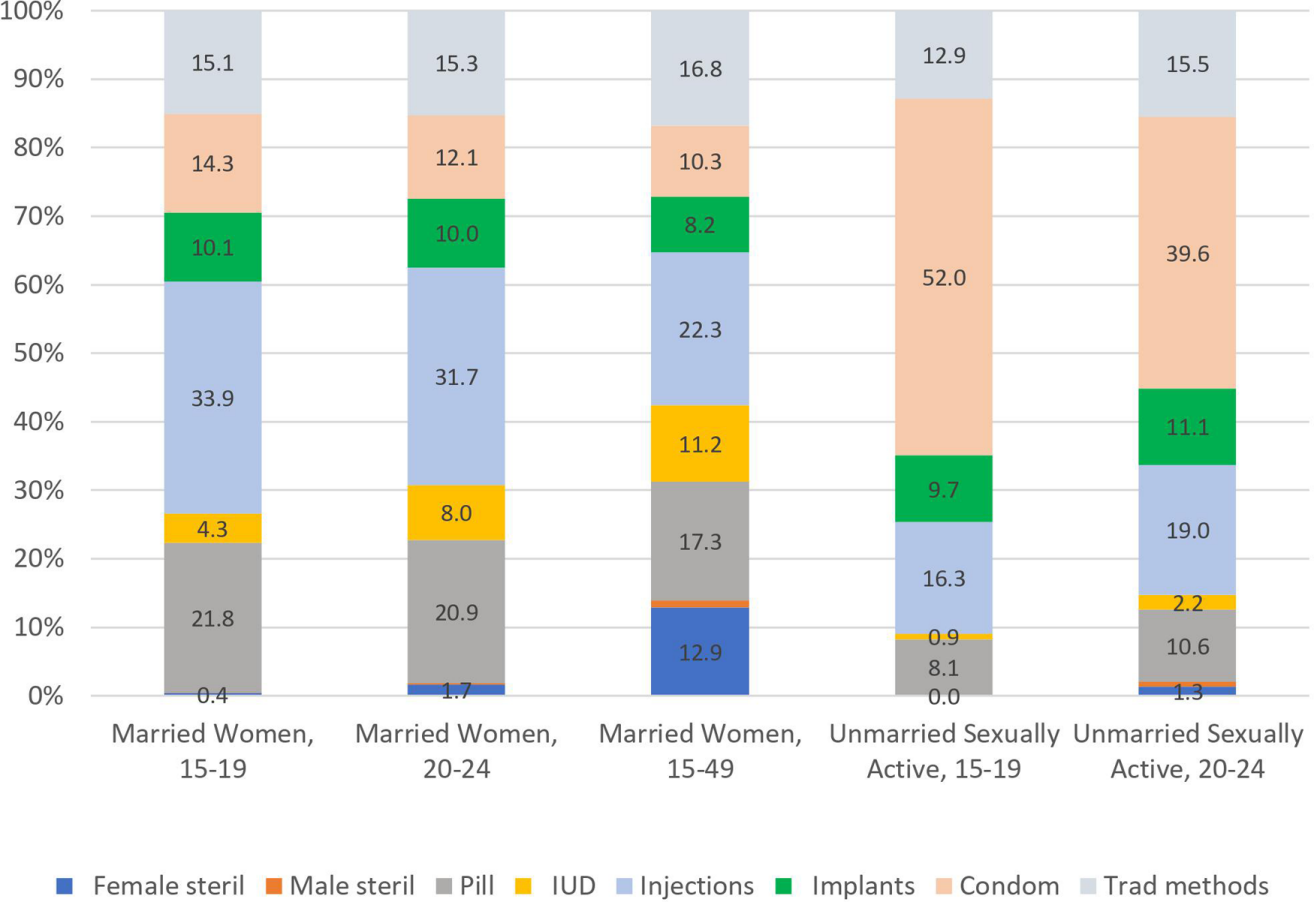
Method Mix by population subgroups, All countries, latest DHS surveys.

The data in Figure 1 provide striking evidence of several major trends in contraceptive method mix in LMICs. First, among married women (displayed on the left-hand side and middle of the figure) method mix is similar for the two young groups aged 15–19 and 20–24 but differs markedly from all married women aged 15–49. Specifically, the younger ages are far more likely to use condoms and injectables, compared to older married women using long-term methods (female sterilization or IUDs). Second, the mix is radically different in the unmarried sexually active group, where the condom dominates at both ages 15–19 and 20–24, compared to young married women (displayed on the righthand side of the figure).

**Figure 2:**
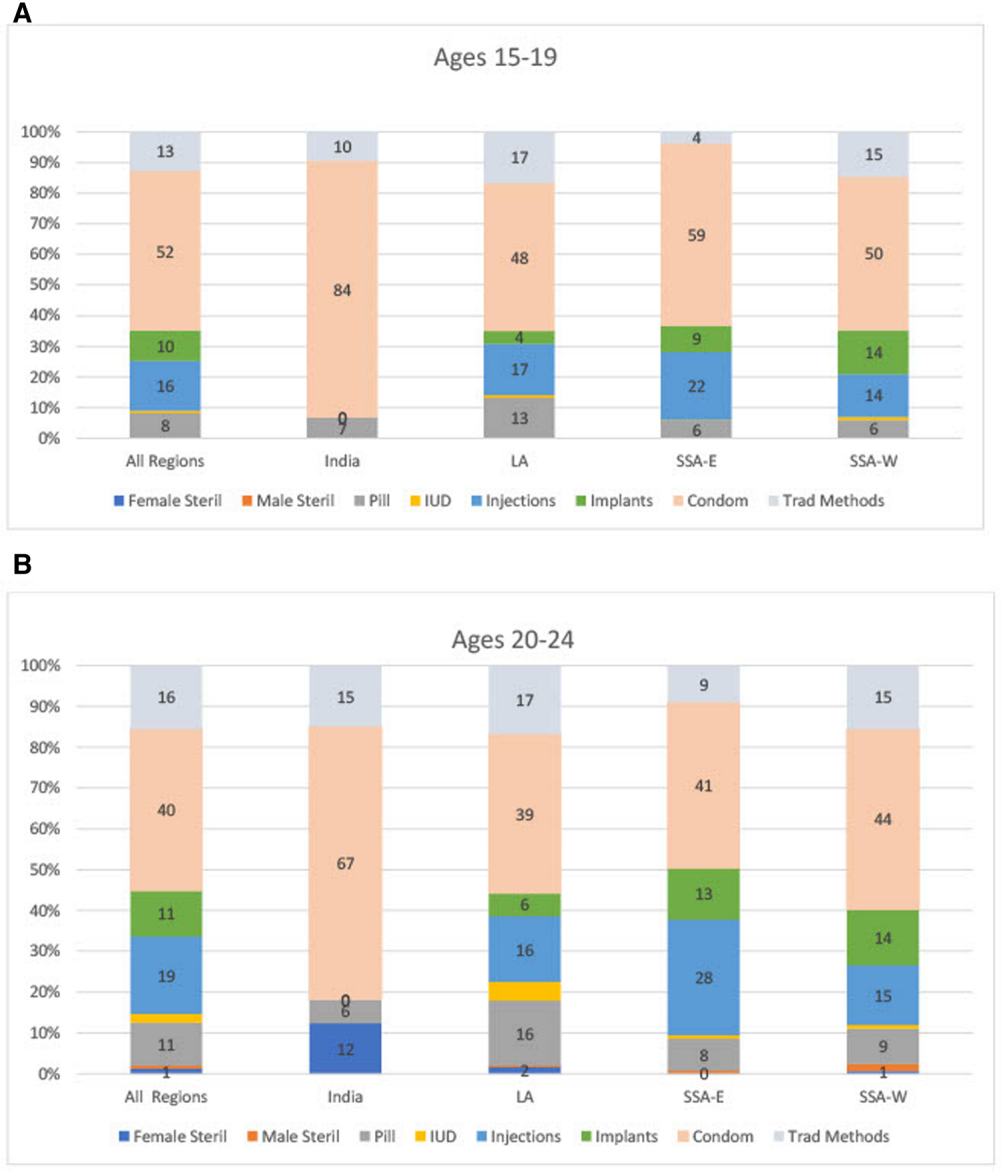
(**A**,**B**) method Mix for unmarried sexually active women, by Age and region.

**Figure 3:**
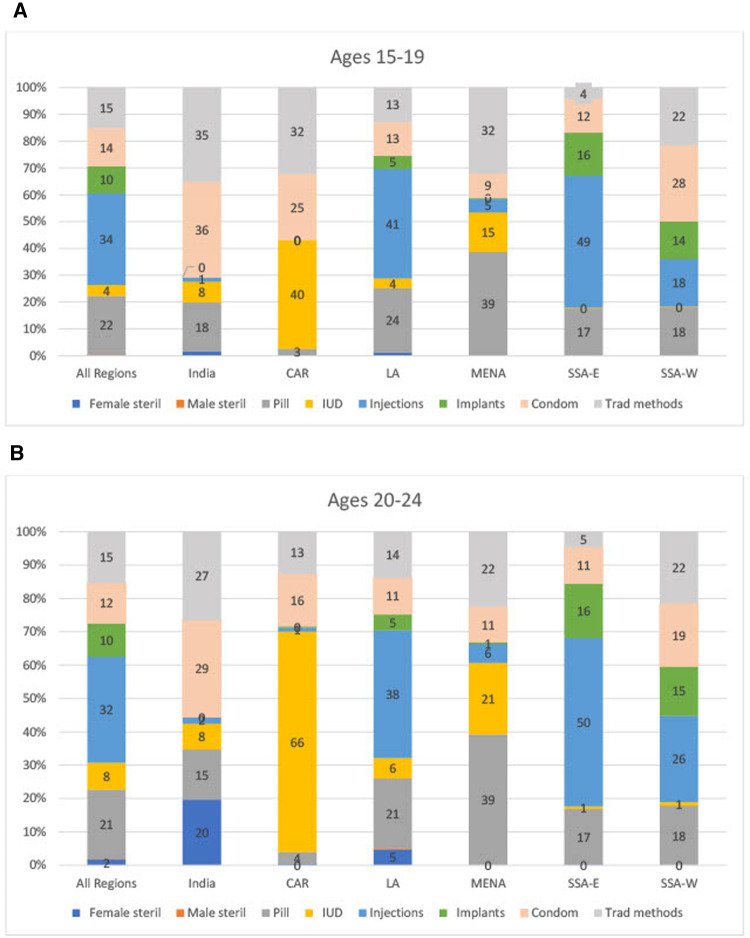
(**A**,**B**) method Mix for married women, by Age and region.

**Figure 4:**
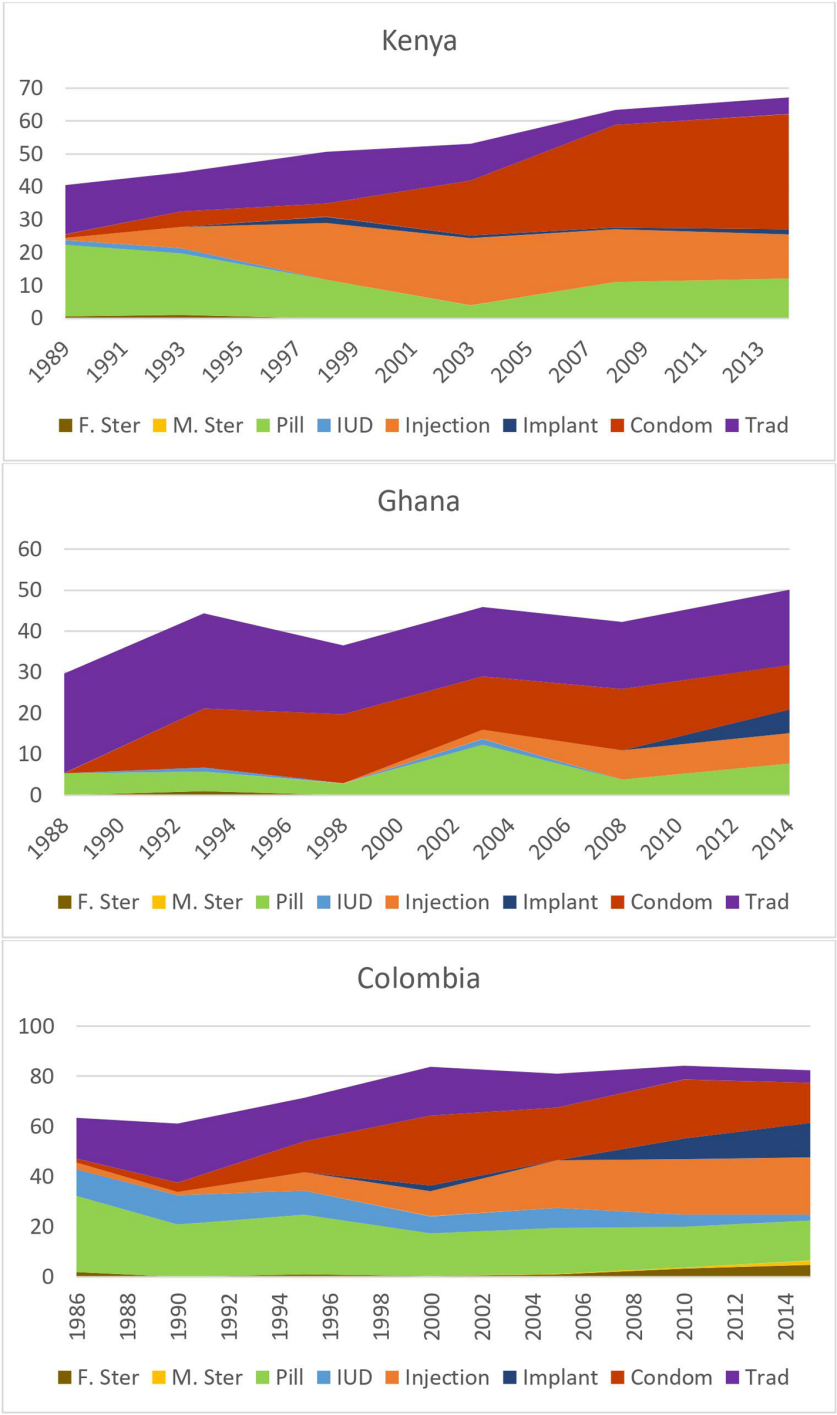
Changes over time in method Mix and contraceptive prevalence Among unmarried sexually active women aged 20–24 in three illustrative countries.

It is important to emphasize that the method mix is based on 100% of users in a given population or sub-population. As such, Figures 1–3 do not measure or reflect levels of contraceptive prevalence among these groups. Whereas one can present both method mix and prevalence in a single graph (as shown in Figures 4), we have retained the “based on 100% use” in Figures 1–3 to allow for comparisons across marital status, age, and region.

### Regional patterns in method mix by marital status and age

We examined regional differences among adolescents and youth along two dimensions: marital status and age.

In terms of marital status, several key trends emerge. For total prevalence of use, unmarried sexually active women report far more contraceptive use than married women of the same age: contraceptive use is 20 percentage points higher in the unmarried sexually active group of women 20–24 (57%) than for married women of the same age (37%) (not shown). Regarding mix, as Figure 1 indicates, unmarried sexually active women are far more likely to use condoms than married women are. Among the unmarried sexually active women condom usage ranged from 40%–52% of the method mix while among married women it ranged from 12%–14%. Married women use a broader range of methods than the unmarried sexually active women do.

By region, LA has the highest level of total contraceptive use (contraceptive prevalence, 73%), followed by SSA-E (57%), with the lowest levels occurring in SSA-W (49%) (not shown). For the method mix for unmarried sexually active women (Figures 2A,B), the mix is relatively similar across regions, as the dominant method remains the condom in India (84% for 15%–19% and 67% for 20–24), LA (48% and 39%) SSA-E (59% and 41%) and SSA-W (50% and 44%).

For married women however, the regional averages are quite different for the method mix (Figures 3A,B, which include both the CAR and MENA regions). Overall, injectables hold the highest share of the method mix at 34% of the mix for 15%–19% and 32% of the mix for 20–24, and this is also the case for LA (41% and 38%), SSA- E (49% and 50%), and SSA-W (18%–26%). But the injectable is nearly absent in India, CAR, and MENA. In CAR, the mix is quite narrow; the IUD is the most popular method among married women (40% and a high 66% for the two ages respectively) while in MENA the pill is the dominant method (39% for both age groups) with a broader overall mix. India is the only country where a sizable percent of women ages 20–24 have had female sterilization, corresponding to 20% of all use. Notably, traditional methods remain important at appreciable levels everywhere except in SSA-E.

Regarding age, the method mix broadens somewhat at ages 20–24 compared to ages 15–19. Across all regions (except married women in MENA), the share of condom usage declines. The mix becomes more balanced with age, especially among unmarried sexually active women. The increased use of more diverse methods suggests a growing awareness of method choice as women age.

### Skewed method mix

We examined both method skew (the yes/no rule wherein a single method represents over half of all use) and method balance using the AD (average deviation) approach. The results are presented first for the rule of half or more of use due to a single method.

In the unmarried sexually active group skew is quite prevalent. At ages 15–19 two-thirds of all countries with data show skew by the one-half rule, reflecting their strong tilt toward condom use. Notably, skew is less in the older, 20–24 group, where less than half (48%) of countries show skew, with relatively less condom use.

Which methods are responsible for skew? To explore this, the average shares held by the leading methods are listed in Table 2 below, based on all unmarried sexually active women (Table 2). Methods representing a lower share of the mix are omitted from this table. Including omitted methods, the sum is 100%. Based on all countries with available data, the condom is dominant in both age groups. The injectable is a low second, along with traditional methods and the implant. Condom use falls off in the older age group.

**Table 2:** Leading methods used by unmarried sexually active women across all LMICs with available data.

	15–19	20–24
Condom	53.5	42.6
Injection	15.9	19.4
Traditional methods	12.9	14.3
Implant	8.8	10.3

Turning to young married women (Table 3) at ages 15–19, over half of countries (54%) show skewed method mixes. For the 20–24 group that is a little less, at 48%. By method there is a close similarity; there is little change in the mix as the women become five years older. In contrast, among all married women of reproductive age, skew is present in 30% of the countries, as found in previous analyses based on a larger number of countries (15).

**Table 3:** Leading methods used by married women by age across all LMICs with available data.

	15–19	20–24	15–49
Injectable	33.2	34.2	25.3
Pill	18.7	19.1	17.1
Traditional methods	17.6	17.0	18.6
Condom	17.9	14.4	11.0

Again, the average shares held by the leading methods help to explain the source of skewed mixes. Below are the results for all three age groups of interest. In the two young married groups the injectable is most prominent with about a third of all users, followed by the other three shown. The injectable share is much less among all women aged 15–49; the full mix is broader, including methods not shown. Condoms rank lowest at ages 20–24 and 15–49 reflecting their declining popularity as women age. The implant corresponds to fewer users overall, but it is important in some countries, especially in the eastern sub-Saharan region (24).

The AD (Average Deviation) method is a second way to examine imbalances in the method mix. Based on the deviations of method shares around the average share (12.5% if all 8 methods are used equally), the AD value is sensitive to variations across all eight methods. Instead of the simpler 50% rule, the value reflects deviations of method levels that fall across a range. For example, some shares can lie well above the 50% cutoff, or just miss it by falling into the high-40s. Levels near zero, as with male sterilization, also contribute to the deviations from the average.

The AD measure has no specific cutoff, as the scores lie along a continuum from low to high, from zero when all shares are equal, to a maximum of 22 when a single method accounts for 100% of all use. As an example, for the unmarried sexually active group aged 20–24 the range is from 8.3 to 19.3, around a mean of 13.9. This varies, affording a way to compare the degree of imbalance over time or across countries.

The basic results follow:

• The imbalance in the mix is greater at ages 15–19 than at ages 20–24, both for unmarried sexually active women and for married women (see Figures 2A,B and 3A-3B, in the “All Regions” bars at the left). For example, for unmarried sexually active women, on the high side condoms have the extreme shares of 52% at ages 15–19 and 40% at ages 20–24. On the low side, male and female sterilization are near zero, in both age groups.
• The imbalance is also greater in the sexually active group than in the married group, in each age comparison. For example, Figure 1 shows for ages 15–19 the large share of 52% for condom use among unmarried sexually active women, against the small share of 14% among married women.
• Among married women, the two young groups each show more imbalance than all women aged 15–49 do. In Figure 1, for all married women ages 15–49, the shares are spread more evenly around the average of 12.5% than they are in the two young age groups. For example, the highest share for all married women is only 22% whereas it is 34% and 32% for the two young groups. The young groups are also near zero for sterilization, whereas the share for all women is at the average (13%).
• The AD values are spread more widely across member countries within the two unmarried sexually active groups than within the married groups. This pertains not to the mix patterns in the figures but rather to variations across countries (not shown). That is, countries differ among themselves in the amount of imbalance, and do so more when we focus on the unmarried sexually active groups than when we focus upon married women. There are fewer extreme values across countries in the mixes for married women.

### Changes in method mix over time among adolescents and young women in three illustrative countries

It is instructive to examine how method mix changes over time, using different countries as examples. In Figure 4, we present data for Kenya (SSA-E), Ghana (SSA-W), and Colombia (LA) for periods of about 25 years, running from the late 1980s to 2013–14 for each country. The choice of countries was limited by the availability of long-term series of data, but these three are broadly representative of the types of changes in other countries in their regions.

Figure 4 shows prevalence of use by method, demonstrating the changes over time, simultaneously with an increase in total contraceptive use among unmarried sexually active women aged 20–24. The key features are:

• The three countries vary from low to high levels in the growth of total contraceptive use for this age group. Colombia has had the most advanced public and private programs, and prevalence was already at 62.6% as early as 1986, rising to a remarkable 84.3%. In Kenya it began at 40.2% and rose steadily over the years to 68.2%, over two thirds of women. Ghana began at only 29.9% and rose to 50.0%.
• The condom has been important historically in all three countries, but especially in Kenya, where 34% of the women are using it. In Ghana it held a large share of total use, ranking second to traditional methods.
• The injectable was very important in both its prevalence and its share in Colombia and Kenya; less so in Ghana.
• The pill too played an important role, followed by a smaller contribution by the implant.
• Traditional methods faded over time in both Colombia and Kenya but remained widespread in Ghana, where the other methods have likely been less available.
• Overall, unmarried sexually active women have turned to a mix of the five contraceptive methods mentioned. The IUD and male and female sterilization have been essentially absent.

The above findings are much different for married women of the same age. Total use is much lower as many women seek a first birth. The condom is far less important, as are traditional methods (except in Ghana). The injectable has been prominent, as well as the pill though with some loss in Colombia and Kenya. Use of the implant has grown in all three countries. The same five methods as above have accounted for nearly all use, in changing proportions.

These cases reflect the three regional settings in terms of their total use and in the reliance on just the five methods. The cases also show the dynamic changes that can occur over the long term, as new methods such as the injectable and implant emerge and as others fade.

## Discussion

A method mix gives the breakdown of methods in actual use in a country. It does not distinguish between what may be the preferred method as opposed to the method chosen among the available options. The various supply or availability constraints, together with the nature of demand for contraception, have produced the method mixes that we see in these national surveys.

Among youth, the greatest difference in the method mixes is between the married and unmarried groups. Fewer married women use any method of contraception, and their method mix is more diverse than that for unmarried sexually active women. Unmarried women of the same age more frequently use condoms, with the pill as a “runner-up.” Since these short-term methods tend to be less effective, with high failure and discontinuation rates, their high utilization contributes to unintended pregnancies. These findings are consistent with previous literature noting the popularity of short-term methods among women aged 15–24 (16, 20, 22).

There is high usage of male condoms among youth and adolescents across all regions. They tend to be provided in both the public and private sectors and are less prone to stockouts than LARCs, making them more widely available than other methods (26). Furthermore, condoms tend to be the least restricted method of contraception compared to more effective methods that providers may discourage based on age or parity. Minimum age bias, for example, involves provider restrictions on family planning methods such as pills or injectables for women under certain ages, which restricts access to these methods for adolescent girls in some countries (25, 26, 27, 28).

Supply side constraints restrict total use and affect the mix; so too do demand constraints. Younger women may elect to use short-acting methods, despite the availability of more effective, longer-term methods. Young women, especially those who are unmarried and sexually active, likely have more sporadic sexual encounters and are less inclined to choose long-acting methods. Furthermore, condom usage may be more acceptable among young unmarried women than young married women since using a condom in marriage can signal distrust between partners. In SSA, the popularity of the condom among youth may also be due in part to the high prevalence of HIV in the region, which has led to the promotion of condoms to prevent infection as well as unwanted pregnancy. These programs have been especially effective at targeting adolescent and young adult populations (29). In general, of the eight methods, male and female sterilization are seldom the choice of youth, and the IUD is little used, especially in SSA. This is consistent with previous literature in SSA, which notes the high use of short-term methods as opposed to long-acting clinical methods (8). Overall, the factors that contribute to the method mixes are highly contextual and specific to the region.

Our analysis found that “method skew” occurs in 30% of LMIC and is much more pronounced among unmarried than for married women. Methods responsible for skew also vary, though the condom tends to dominate the skew patterns among adolescents. A skewed method mix generally indicates limited method choice. In many countries, method skew has been taken as a call for corrective program actions. The picture is somewhat complex; interestingly, skew does not correlate strongly with total use (15). Countries such as Vietnam and China have high levels of use in the presence of heavy reliance on one or two methods, for example. Moreover, for the unmarried sexually active women, highly skewed method distributions can be understandable, reflecting their strong preference for condoms. Measures of skew are best seen as neutral indications of imbalance in the mix (4). However highly skewed method mixes should be a red flag for further investigation. They may reflect personal or cultural preferences but they are often the result of issues such as contraceptive availability and provider biases.

The mixes vary in which methods are responsible for imbalances: that is a constant theme across ages, marital statuses, regions, and countries. Programmatic actions to expand contraceptive choice and extend it to more countries must necessarily follow the patterns of these variations. Countries or regions may face constraints in expanding the method mix, for example with permanent contraception in Muslim societies. Similarly, permanent contraception is not widely accepted in SSA countries, because of a desire to maintain the possibility of producing another child even if one has met one's desired number of children. Countries that lack hygienic conditions in rural facilities may steer away from IUD insertions. Nevertheless, some expansion of choice at the margins of these limitations is possible and can be among programmatic options for youth of both marital statuses. Where the pill, injectable, or implant is not readily available, access to it can be improved, at least in some settings.

The usage of long-acting reversible contraceptives (LARCs) remains low among young populations across almost all regions (except in the CAR region). This is due in part to societal expectations for recently married women to obtain contraception only after the first birth has occurred (25). In these situations, demand generation may be a useful intervention for improving uptake or introducing more effective, long-term methods. Younger women have been increasingly delaying childbearing, which also may contribute to the overall demand generation for LARCs in the future (30). On the supply side, younger women tend to seek care from limited-capacity and informal providers due to the embarrassment or stigma involved in obtaining family planning (31). Short-acting and non-clinical methods are more readily available in these settings, so interventions that seek to strengthen the capacity of such providers may improve the overall availability of a larger range of contraceptives.

Our analysis of the evolution of method mix over time is limited to three illustrative countries. Over a 25-year period, these countries show how the mix can respond to the introduction of new technology and its increased availability. The trends also show how the displacement of one method by others can occur while the total prevalence of use is rising. This is especially true in the case of the injectable across all three countries; as the method appeared on the market and became more highly available, its share of the method mix has increased, and the overall prevalence of use rose. Such developments impact the choices available for youth.

The current study is not without limitations. The article focuses only on women, as males were excluded for the lack of available data from comparable multi-national surveys. In interviews, unmarried males would likely report more condom usage than unmarried women would, though the two would probably be closer among married couples, as supported by previous findings (30, 31). Furthermore, the analysis is limited to the series of DHS surveys, as the only large set of information on contraceptive use by unmarried sexually active women. We implemented a cutoff of 2000 for DHS survey inclusions; within that, 54 of the 64 surveys occurred after 2010. About half of the surveys are from sub-Saharan Africa, divided nearly equally between the East/South and West/Central regions. This selection of countries is by no means representative of LMIC worldwide. Our analysis used unweighted data by country, which gives equal weight to large and small nations. Our analysis also did not analyze data regarding women's overall satisfaction with their current method, nor whether they were able to obtain their preferred method.

We would expect the method choices for youth and adolescents to change as they age and move into new life stages, with enlarging family sizes. These lifecycle changes can affect their demand for contraception and their preferences for methods, shifting toward more certain, long-term methods. We find rather modest changes between the two youngest age groups, but the patterns are much different between the unmarried and the married groups. The findings are important regarding the method mixes of youth and adolescent populations across regions and marital status, reflecting the wide variations that call for local program adaptations.

From a historical perspective, the substantial amount of contraceptive use by unmarried sexually active women is a relatively recent development. We did not discuss the rising age of marriage (32, 33), but it, together with the adoption of contraceptive methods by both unmarried and married women, signals an improvement in the empowerment of both groups. Given that consistently inferior quality of care has been reported by younger women (31), improvements to the quality of care can occur by expanding method choice among youth, and integrating adolescent-friendly services into family planning programming (25). The current study, overall, finds promising trends in overall contraceptive uptake among youth that can be built upon to improve health outcomes for young women.

## Data Availability

Publicly available datasets were analyzed in this study. This data can be found here: https://www.statcompiler.com/en/.
